# WHO IS THAT MAN I SEE STARING STRAIGHT BACK AT ME? - Mirror Delusional Misidentification: A Case Report And Literature Review

**DOI:** 10.1192/j.eurpsy.2022.2267

**Published:** 2022-09-01

**Authors:** S. Mouta, I. Fonseca Vaz, B. Jesus, J. Correia, S. Fontes

**Affiliations:** Unidade Local de Saúde da Guarda, Departamento De Psiquiatria E Saúde Mental, Guarda, Portugal

**Keywords:** Neuropsychiatry, Mirror Delusional Misidentification, Delusional Misidentification Syndromes

## Abstract

**Introduction:**

The delusional misidentification syndromes (DMS) are uncommon but fascinating neuropsychiatric disorders. One particularly intriguing form of DMS is called the mirror sign or mirror delusional misidentification (MDM).

**Objectives:**

We aim to present a case on MDM and a review on MDM and its correlation with neurological lesions.

**Methods:**

Non-systematic review of the literature and case report.

**Results:**

A 72 years old patient was admitted to the emergency department with disorientation, behavioral changes and persecutory delusional ideation. The patient was also unable to recognize his face in the mirror, claiming to be his son. On neuroimaging tests, the patient presented with moderate diffuse cortical-subcortical cerebral atrophy associated with mild diffuse cortical cerebellar atrophy, as well as atheromatous calcifications in carotid siphons. In the MDM, the patient treats the mirror image as separate from the self. It is commonly seen in patients with dementia. Unlike Capgras syndrome, MDM is typically associated with neurological illness, particularly with neurodegenerative conditions. Findings on neuroimaging have shown a pattern of right hemisphere cortical and subcortical lesions. the most common findings included the following: generalized or localized atrophy on Magnetic Resonance Imaging, ventricular dilatation on Computed Tomography scan, and slowing on Electroencephalography.

**Conclusions:**

Mirror delusional misidentification differs from other forms of DMS as it is seen exclusively in patients with neurological disease. While right hemisphere dysfunction appears to be a requirement for MDM, patients with this condition do not show consistent enough neuroimaging findings to allow for a localization within the right hemisphere.

**Disclosure:**

No significant relationships.

